# ^1^H‐NMR‐based metabolomics reveals the preventive effect of *Enteromorpha prolifera* polysaccharides on diabetes in Zucker diabetic fatty rats

**DOI:** 10.1002/fsn3.4061

**Published:** 2024-03-05

**Authors:** Jie Chen, Shuting Wang, Fuchuan Guo, Yupeng Gong, Tianbao Chen, Chris Shaw, Rencai Jiang, Fang Huang, Dai Lin

**Affiliations:** ^1^ Department of Nutrition and Food Safety, School of Public Health Fujian Medical University Fuzhou Fujian China; ^2^ School of Pharmacy Queen's University Belfast UK

**Keywords:** branched‐chain amino acid, *Enteromorpha prolifera* polysaccharides, glycemic abnormalities, metabolomics

## Abstract

The primary objective of this investigation was to explore the beneficial impacts of *Enteromorpha prolifera* polysaccharide (EP) on dysglycemia in Zucker diabetic fatty (ZDF) rats, while also shedding light on its potential mechanism using ^1^H‐NMR‐based metabolomics. The results demonstrated a noteworthy reduction in fasting blood glucose (FBG, 46.3%), fasting insulin (50.17%), glycosylated hemoglobin A1c (HbA1c, 44.1%), and homeostatic model assessment of insulin resistance (HOMA‐IR, 59.75%) following EP administration, while the insulin sensitivity index (ISI, 19.6%) and homeostatic model assessment of β‐cell function (HOMA‐β, 2.5‐fold) were significantly increased. These findings indicate that EP enhances β‐cell function, increases insulin sensitivity, and improves insulin resistance caused by diabetes. Moreover, EP significantly reduced serum lipid levels, suggesting improvement of dyslipidemia. Through the analysis of serum metabolomics, 17 metabolites were found to be altered in diabetic rats, 14 of which were upregulated and 3 of which were downregulated. Notably, the administration of EP successfully reversed the abnormal levels of 9 out of the 17 metabolites. Pathway analysis further revealed that EP treatment partially restored metabolic dysfunction, with notable effects observed in valine, leucine, and isoleucine metabolism; aminoacyl‐transfer RNA (tRNA) biosynthesis; and ketone body metabolism. These findings collectively indicate the potential therapeutic efficacy of EP in preventing glycemic abnormalities and improving insulin resistance. Thus, EP holds promise as a valuable treatment option for individuals with diabetes.

## Graphical abstract

This study aimed to investigate the ameliorative effects of *Enteromorpha prolifera* polysaccharide (EP) on Zucker diabetic fatty (ZDF) rats and elucidate its potential mechanism using ^1^H‐NMR‐based metabolomics. We found that EP has potential as a therapeutic agent for preventing glycemic abnormalities and improving insulin resistance, thereby suggesting its utility in the treatment of diabetes.

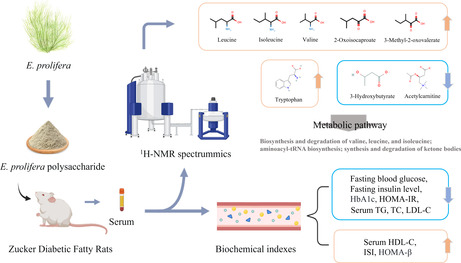

## 1 INTRODUCTION

As a prevalent metabolic syndrome, type 2 diabetes (T2D) is characterized by disrupted glucose homeostasis, resulting in abnormal blood glucose levels (Rines et al., 2016). It affects a significant portion of the global population, with an estimated 416 million adults worldwide living with diabetes in 2017, approximately 90% of whom are diagnosed with T2D (Saeedi et al., 2020). It is anticipated that by the year 2035, there will be an estimated 180 million new cases of diabetes, with a gradual increase in disease prevalence among the younger demographic (Ramzan et al., 2022). In addition, T2D significantly exacerbates the risk of other chronic health issues, including cardiovascular, kidney, and skin diseases (Saeedi et al., 2020), and is a significant global public health concern. Oral hypoglycemic drugs are commonly used for managing blood glucose levels, but they have limitations and drawbacks, such as the need for long‐term treatment and the potential for gastrointestinal side effects (Kato et al., 2020). Polysaccharide is a kind of natural macromolecule that is widely found in plants (including herbs and algae). In recent years, it has attracted increased amounts of attention due to their favorable properties, which include lowering reactive oxygen species (ROS) production, modifying the composition of intestinal microbes, and regulating the secretion spectrum of inflammatory factors (He et al., 2018; Wu et al., 2019; Yang et al., 2019). Exploration and development of edible natural polysaccharides for the prevention and treatment of T2D holds significant promise in combating the current diabetes epidemic.

*Enteromorpha prolifera* (known as *Ulva*), a marine green algal species found in coastal areas of China, has a long history of use as a food source and traditional herb for the treatment of various ailments, including heat dissipation and hydropic diseases (Tang et al., 2013). Previous research has indicated that *Enteromorpha* species are rich in carbohydrates and proteins but have a low fat content (Yu et al., 2017). Structural analysis revealed that EP is a sulfated polysaccharide with sulfate groups commonly attached to the C‐3 position of rhamnose and the C‐2 position of xylose (Chi et al., 2020; Han et al., 2021), which supports its biological activity. EP has been recognized for its various biological effects, such as anti‐inflammatory, anticoagulant, antioxidant, antitumor, and hypolipidemic activities (Tang et al., 2013). Nevertheless, the antidiabetic effect and underlying mechanism of EP have not been fully evaluated, especially the tracking of metabolic processes and their correlation with T2D. To assess the antidiabetic effects of EP, Zucker diabetic fatty (ZDF) rats with the *fa/fa* and *fa/+* genotypes were used in this work. Metabolomic analysis based on ^1^H NMR spectroscopy was conducted to identify biomarkers and investigate the influence of EP on bodily metabolism. The results obtained from this study hold promising implications for the practical utilization of polysaccharides and establish a foundation for future investigations into the antidiabetic mechanisms of EP.

## 2 MATERIALS AND METHODS

### 2.1 Materials

*Enteromorpha prolifera* powders were purchased from Fujian Haixing Health Food Co., Ltd (Fuqing, China). Six‐week‐old obese Zucker diabetic fatty (ZDF) male rats with the *fa/fa* genotype and lean ZDF male rats with the *fa/+* genotype were provided by Weitong Lihua Experimental Animal Technology Co., Ltd (Beijing, China). Anachro‐certified deuterium oxide sodium salt standard solution (ACDSS) was procured from Anachro Technologies Inc. (Wuhan, China). Commercial kits for biochemical assays, including triglyceride (TG, detection range: 0.05–9.0 mmol/L), total cholesterol (TC, detection range: 0–19.39 mmol/L), high‐density lipoprotein cholesterol (HDL‐C, detection range: 0–5.16 mmol/L), and low‐density lipoprotein cholesterol (LDL‐C, detection range: 0–10.4 mmol/L), were obtained from Jiancheng Bioengineering Institute (Nanjing, China). Serum insulin enzyme‐linked immunosorbent assay (ELISA) kits (detection range: 3–200 mU/L) and glycosylated hemoglobin A1c (HbA1c) ELISA kits (detection range: 1.563–100 ng/mL) were purchased from Fine Biotech Co., Ltd (Wuhan, China). The sensitivity of the commercial kit is shown in Supplementary Materials Table S1.

### 2.2 Extraction and purification of EP

The crude polysaccharide was extracted from *E. prolifera* powders using the water extraction–alcohol precipitation technique, as previously described (Zhao et al., 2022). In brief, 100 g of powder was extracted with water at 80°C for 2 h. The extraction solution was centrifuged at 3000× *g* for 10 min, after which the supernatant was precipitated by adding a fourfold volume of ethanol. After repeating the operation twice, the precipitate was dissolved, centrifuged, and filtered through a 0.45‐μm microporous filter membrane. The crude extract was then injected into a DEAE‐52 column, and gradient elution was performed using 0 ~ 1.0 mmol/L NaCl solution. The eluent was collected, injected into a Sephadex G‐100 column, and subsequently eluted with 0.05 mmol/L NaCl, as described in a previous study (Ren, Gong, et al., 2018). The purified EP was obtained after lyophilization for 10 h.

### 2.3 Characterization of EP

The total carbohydrate content of the EP was determined by the phenol–sulfuric acid colorimetric method (Masuko et al., 2005) using D‐glucose as a standard at 490 nm. The protein content was measured using Bradford's method (Bradford, 1976). The monosaccharide compositions were analyzed by gas chromatography (Shimadzu, Japan) using an OV‐101 capillary column (30 m × 0.25 mm ID). The sample pretreatment method and the conditions of the column oven were programmed according to previously reported methods (Xu et al., 2015). Monosaccharide identification was performed by comparison with the standard substances (rhamnose, xylose, mannose, glucose, and galactose).

### 2.4 Animal experiments

The animal experiments were carried out in accordance with the Animal Care and Use Committee of Fujian Medical University (Approval No. FJMU 2021‐0012). A total of 20 obese ZDF rats and 10 lean ZDF rats were housed in a regulated setting, kept at a temperature of 23 ± 2°C, and fluctuating relative humidity between 55 ± 5%, and subjected to a 12‐hour cycle of light and darkness (Yuan et al., 2018). The rats were given unrestricted access to standard rodent feed and normal water. Before being grouped, all rats underwent a one‐week acclimation period to the laboratory conditions. Then, 20 obese ZDF rats were randomly allocated into two groups (*n* = 10) in the following manner: the EP intervention group (EP) received EP through gavage at a dosage of 200 mg/kg body weight and the diabetic model group (DM) received an equal volume of distilled water through gavage. Ten lean Zucker rats (*fa/+*) were used as a control group (NC) and received distilled water via intragastric administration. To establish and evaluate a diabetic model, all rats were fed a Purina #5008 diet, which provided 4.15 kcal/g of energy with 56.4 kcal% carbohydrate, 26.9 kcal% protein, and 16.7 kcal% fat. The chemical composition of Purina #5008 is displayed in Supplementary Materials Table S2. The dose of EP used in this study was determined based on previous animal experiments (Ren, Yang, et al., 2018). Food intake was assessed daily, and the feed was replaced every 3 days to prevent fat oxidation. All rats were weighed twice a week, and the weight gain was recorded to calculate the food efficiency ratio using the following formula (Saleh Gazwi & Mahmoud, 2019):

$$\text{Food efficiency ratio}=\frac{\text{Body weight gain}\;\left(g\right)}{\text{food intake}\;\left(g\right)}$$

### 2.5 Biochemical assays

During the feeding procedure, recent blood samples were obtained from the tail vein to observe fasting blood glucose (FBG) levels using a GA‐6 glucose meter (Sinocare, China) on a weekly basis. After a 12‐hour period of fasting, an oral glucose tolerance test (OGTT) was conducted at the conclusion of the nine‐week intervention period. The rats were administered an oral dose of 2 g/kg glucose, and blood samples (20 μL) were collected at 0, 30, 60, and 120 min for examination. The area under the curve (AUC) of the OGTT was calculated using the trapezoidal method, as described in previous reports (Zhang et al., 2020). After the experiment, the rats were fasted for 12 h, and blood samples were taken from the retro‐orbital venous plexus under isoflurane (3.5%) anesthesia. Then, the blood was centrifuged at 3000× *g* for 10 min (at 4°C) to obtain the serum for biochemical analysis. Serum lipid parameters were assessed using commercially available kits and examined on a Multiskan microplate reader (Thermo Fisher, USA). Fasting serum insulin and serum HbA1c were measured using ELISA kits according to the manufacturer's protocol. The HOMA‐IR, HOMA‐β, and ISI were calculated using the following formulas (Chen et al., 2016):

$$\text{HOMA}-\mathrm{IR}=\frac{\text{fasting insulin}\;\left(\mathrm{\mu U}/\mathrm{mL}\right)\times \text{fasting blood glucose}\;\left(\text{mmol}/L\right)}{22.5}$$

$$\text{HOMA}-\beta =\frac{20\times \text{fasting insulin}\;\left(\mathrm{\mu U}/\mathrm{mL}\right)}{\text{fasting blood glucose}\;\left(\text{mmol}/L\right)-3.5}$$

$$\mathrm{ISI}=\mathrm{Ln}\;\frac{1}{\text{fasting insulin}\;\left(\mathrm{\mu U}/\mathrm{mL}\right)\times \text{fasting blood glucose}\;\left(\text{mmol}/L\right)}$$

### 2.6 Metabolite extraction procedure

To extract metabolites, each serum sample was centrifuged at 13,000 rpm for 2 min at 4°C. The resulting supernatant was then transferred to a 3‐kDa ultrafiltration filter (Millipore, Massachusetts, USA). After an additional centrifugation step at 13,000 revolutions per minute (rpm) for 45 min, 480 μL of the filter solution was collected and mixed with 50 μL of DSS standard solution. The resulting mixture was mixed and centrifuged at 13,000 rpm. Finally, 480 μL of the supernatant was transferred to a 5 mm NMR tube (Norell, Morgantown, USA) for collection of the nuclear magnetic resonance (NMR) spectrum.

### 2.7 ^1^H NMR spectrometry

All the samples were subjected to analysis using a Bruker Avance III HD 600 MHz NMR spectrometer (Bruker, Karlsruhe, Germany). The first increment of the noesypr1d pulse sequence was employed for the acquisition of ^1^H‐NMR data and for attenuating the solvent signal. The experiments utilized a 100 ms mixing time in combination with a 990 ms pre‐saturation. The chemical shifts of all the spectra were referenced to the ACDSS peak at 0.0 ppm, serving as the standard. Spectra were gathered at 25°C, with a grand total of 128 scans spanning a duration of 15 min (Chen et al., 2020). The peak shape of the spectra was adjusted through the inversion convolution operation.

### 2.8 Data processing and multivariate analysis

The gathered free induction decay (FID) signal was automatically expanded with additional data points and subjected to Fourier transformation within the Processing module of Chenomx NMR Suite version 8.1. (Chenomx Inc., Edmonton, Canada). The data were subsequently adjusted for phase and baseline using the Chenomx Processor. A total of 50 metabolites were identified and quantified from ^1^H NMR spectra by comparing with the Chenomx database (Xiao et al., 2019). The absolute concentration of all metabolites was exported to Excel and normalized by weight across all parallel samples before used in the subsequent multivariable analysis.

Principal component analysis (PCA) and partial least squares discriminant analysis (PLS‐DA) were employed for visualizing the distribution trend, utilizing the OmicStudio tools (Lianchuang, Hangzhou, China). Differential metabolites were identified based on a screening condition of variable importance in projection (VIP) >1 and an independent sample *t*‐test with a significance threshold of *p* < .05. Metabolites with an impact value>0.2 were considered potential biomarkers (Li et al., 2019) for further investigation. Differential metabolites were estimated by metabolic pathway analysis using MetaboAnalyst version 4.0 (https://www.metaboanalyst.ca/) based on the KEGG (Kyoto Encyclopedia of Genes and Genomes) database.

### 2.9 Statistical analysis

Chemical composition analysis of EP was performed in triplicate, and the data were presented as the mean ± standard deviation (SD). The data for rats (*n* = 8–10) were expressed as the mean ± SD or visualized analysis using GraphPad Prism (GraphPad Software, USA). Fisher's least significant difference (LSD) test in SPSS 20.0 software (IBM SPSS, USA) was used to statistically analyze the differences among the experimental groups. Spearman's correlation clustering heatmap was utilized to visualize between the differential metabolites and physiological index through the OmicStudio Cloud Platform (https://www.omicstudio.cn). Any test result with a value of *p* < .05 was considered statistically significant.

## 3 RESULTS

### 3.1 Characterization of EP

As shown in Table 1, the total carbohydrate content of the EP was 76.3% and the protein content was 1.8%. The results of the monosaccharide composition analysis showed that EP was a heteropolysaccharide composed of rhamnose, xylose, glucose, galactose, and mannose, with a molar ratio of 21.2:5.5:2.9:1:0.9. This indicates that EP is a typical rhamnose‐rich polysaccharide, which is consistent with the structural characteristics of similar algal polysaccharides (Zhong et al., 2020).

**TABLE 1:** Chemical composition and monosaccharide analysis of EP.

Sample	Total carbohydrate content (%)	Protein content (%)	Monosaccharide (molar ratio)				
			Rha	Xyl	Man	Glu	Gal
EP	76.3 ± 2.7	1.8 ± 0.2	21.2	5.5	0.9	2.9	1.0

### 3.2 Effects of EP on body weight, organ mass, food intake, and the food efficiency ratio

As illustrated in Table 2, the initial body weight did not show notable variation among the three groups. However, after 9 weeks, the final body weight, liver mass, epididymal fat mass, and perirenal fat mass of the DM group exhibited significant increases compared to those of the NC group. In contrast, the pancreatic mass in the DM group was markedly lower than that in the NC group. Compared with the DM group, the final body weight of the EP group showed a decreasing trend, although the difference was not significant. Notably, EP administration markedly decreased perirenal fat mass, and markedly increased pancreatic mass. However, there were no significant alterations observed in the liver mass and epididymal fat mass between the EP and DM groups. In terms of food consumption, there was no significantly obvious difference in food efficiency among the three groups, while the average food intake in the DM group was significantly higher than that in the NC group during the experimental period.

**TABLE 2:** Effects of EP on body weight, organ mass, food intake, and food efficiency ratio.

	NC group	DM group	EP group
Initial body weight (g)	180.5 ± 8.2	193.6 ± 7.1	191.4 ± 5.9
Final body weight (g)	311.2 ± 17.1	410.1 ± 20.8^#^	383.9 ± 32.1
Liver mass (g/100 g BW)	2.78 ± 0.13	4.66 ± 0.28^#^	4.55 ± 0.27
Pancreatic mass (g/100 g BW)	0.35 ± 0.06	0.21 ± 0.05^#^	0.31 ± 0.06*
Epididymal fat mass (g/100 g BW)	0.54 ± 0.06	0.96 ± 0.18^#^	0.90 ± 0.07
Perirenal fat mass (g/100 g BW)	0.51 ± 0.12	1.65 ± 0.19^#^	0.97 ± 0.11*
Food intake (g/day)	20.4 ± 2.7	34.8 ± 3.9^#^	29.7 ± 4.4
Food efficiency ratio	0.151	0.154	0.153

*Note*: The data are presented in the form of the mean ± SD. NC: control group (*n* = 9); DM: type 2 diabetic model group (*n* = 8), EP: intervention group (*n* = 10), gavaged with 200 mg/kg body weight EP.

^#^ *p* < .05 compared with the NC group;

* *p* < .05 compared with the DM group.

### 3.3 Effects of EP on serum biochemical indices

**FIGURE 1:**
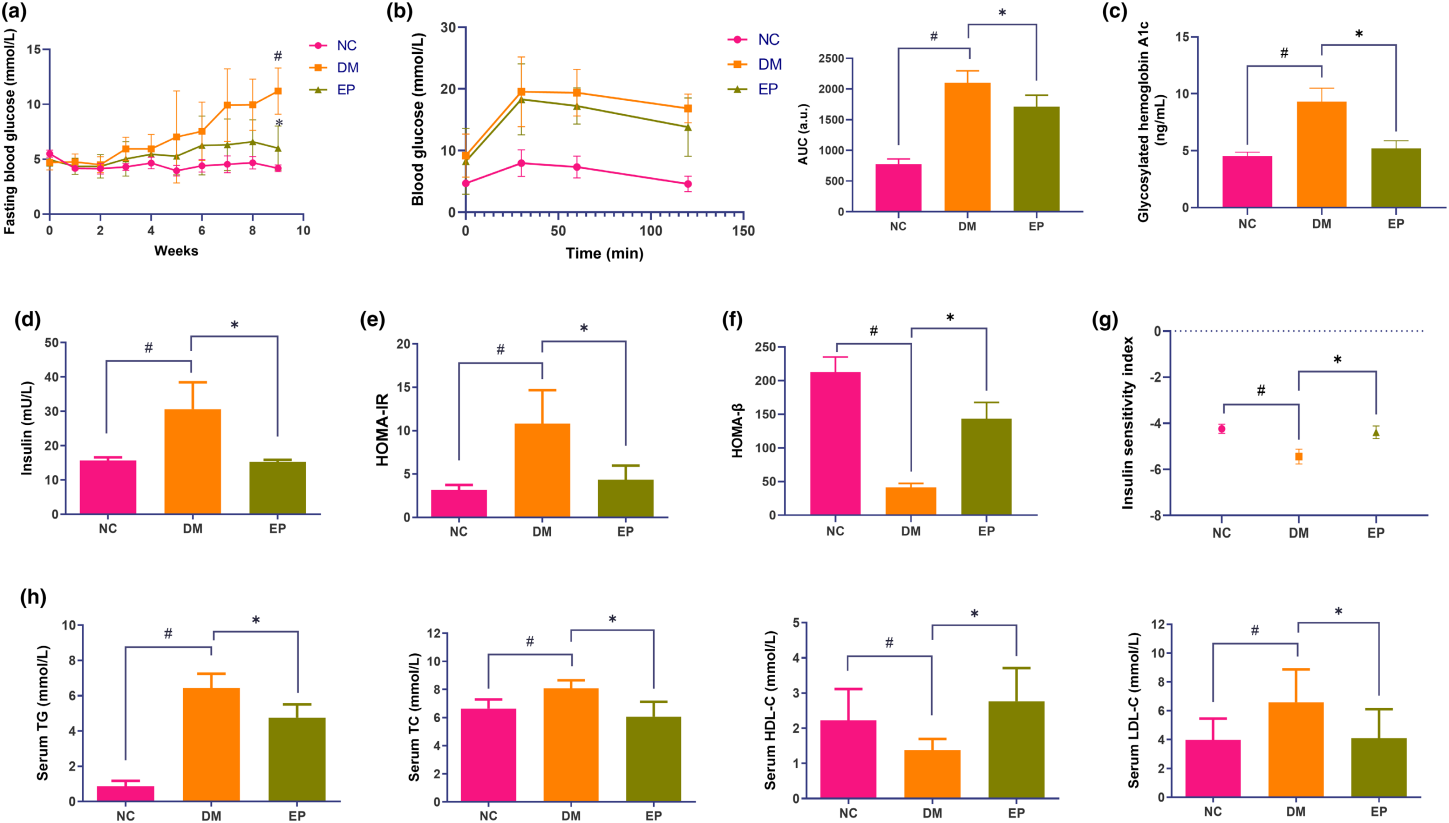
Effects of EP on biochemical parameters in ZDF rats. (a) Changes in fasting blood glucose over 9 weeks. (b) Curve and the area under the curve (AUC) of the oral glucose tolerance test (OGTT). (c) Glycosylated hemoglobin A1c (HbA1c). (d) Fasting serum insulin levels. (e) Homeostasis model assessment of insulin resistance (HOMA‐IR). (f) Homeostatic model assessment of β‐cell function (HOMA‐β). (g) Insulin sensitivity index (ISI). (h) Serum lipid profile. NC: control group (*n* = 9); DM: type 2 diabetic model group (*n* = 8), EP: intervention group (*n* = 10), gavaged with 200 mg/kg body weight EP. ^#^ *p* < .05 compared with the NC group; *p < .05 compared with the DM group.

Throughout the intervention period, the FBG levels remained stable in the NC and EP groups, while those in the DM group showed an upward trend. At the conclusion of the ninth week, the FBG levels in the DM group reached 11.20 ± 2.11 mmol/L, representing a marked increase in comparison to the levels observed in the NC group (4.17 ± 0.30 mmol/L). The FBG level of the rats in the EP group was 6.01 ± 2.02 mmol/L (Figure 1a), which was markedly lower than that of the DM group. Additionally, the results of the OGTT indicated a significant increase in the area under the curve (AUC) in the DM group compared to that in the NC group. The administration of EP resulted in a noteworthy 18.6% reduction in the AUC (Figure 1b). Notably, the DM group had elevated fasting insulin level, HbA1c level, and HOMA‐IR, while the EP group had reductions of 50.17%, 44.1%, and 59.75%, respectively (Figure 1c–e). In contrast, compared with those in the DM group, the EP group exhibited a 2.5‐fold increase in HOMA‐β, and a 19.6% increase in ISI (Figure 1f,g). Furthermore, the EP group showed significant differences in serum lipid profiles (Figure 1h) compared with the DM group, characterized by lower TG (26.1%), TC (25.1%), and LDL‐C (37.9%), while higher levels of HDL‐C (1.0‐fold) levels.

### 3.4 Multivariate analysis

**FIGURE 2:**
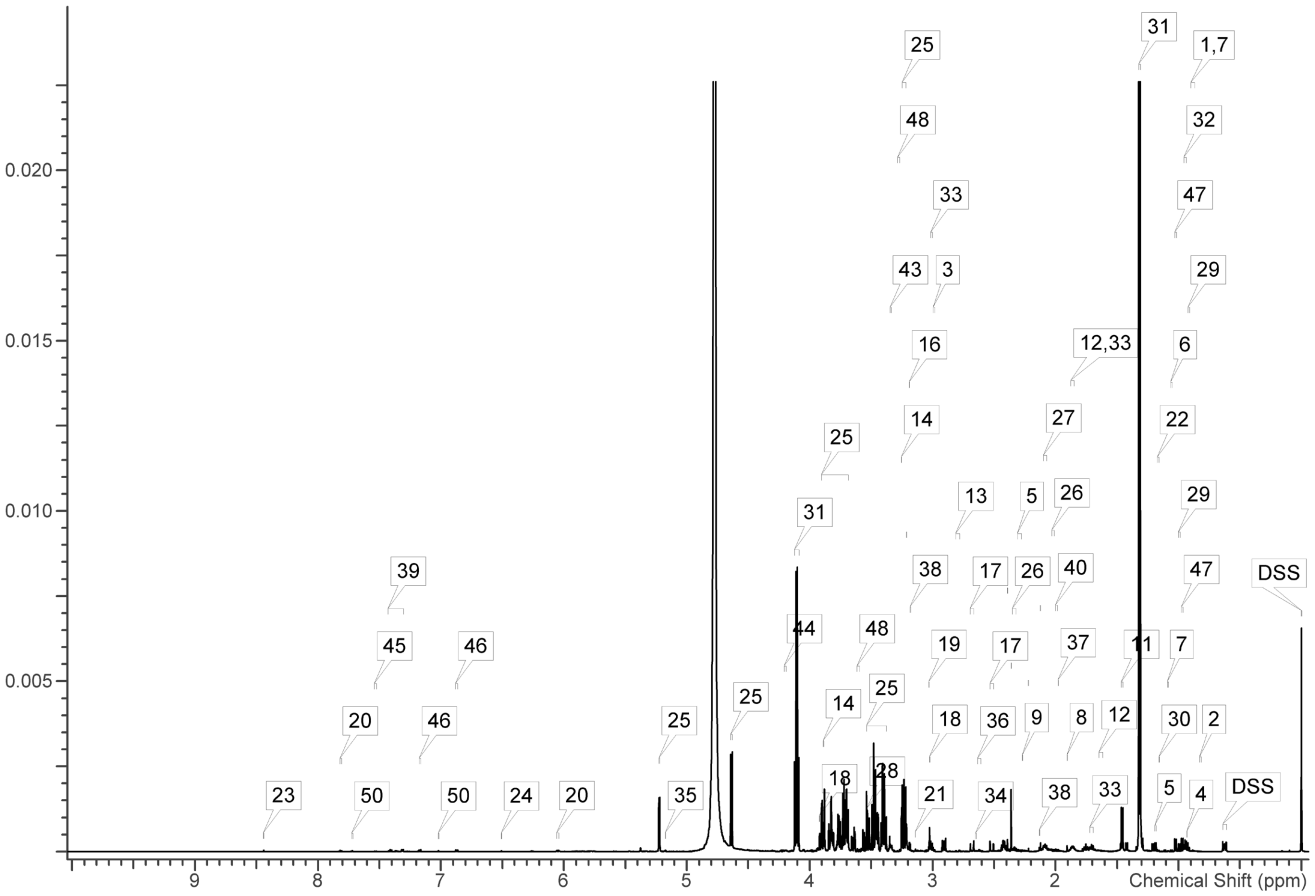
Representative ^1^H‐NMR spectrum (0–10 ppm) for the detected metabolites in a serum sample. (1) 2‐Hydroxybutyrate; (2) 2‐Hydroxyisovalerate; (3) 2‐Oxoglutarate; (4) 2‐Oxoisocaproate; (5) 3‐Hydroxybutyrate; (6) 3‐Hydroxyisobutyrate; (7) 3‐Methyl‐2‐oxovalerate; (8) Acetate; (9) Acetoacetate; (10) Acetone; (11) Alanine; (12) Arginine; (13) Aspartate; (14) Betaine; (15) Carnitine; (16) Choline; (17) Citrate; (18) Creatine; (19) Creatinine; (20) Cytidine; (21) Dimethyl sulfone; (22) Ethanol; (23) Formate; (24) Fumarate; (25) Glucose; (26) Glutamate; (27) Glutamine; (28) Glycine; (29) Isoleucine; (30) Isopropanol; (31) Lactate; (32) Leucine; (33) Lysine: (34) Malate; (35) Mannose; (36) Methionine; (37) N6‐Acetyllysine; (38) O‐Acetylcarnitine; (39) Phenylalanine; (40) Proline; (41) Pyruvate; (42) Succinate; (43) Taurine; (44) Threonine; (45) Tryptophan; (46) Tyrosine; (47) Valine; (48) Myo‐inositol; (49) sn‐Glycero‐3‐phosphocholine; (50) τ‐Methylhistidine.

**FIGURE 3:**
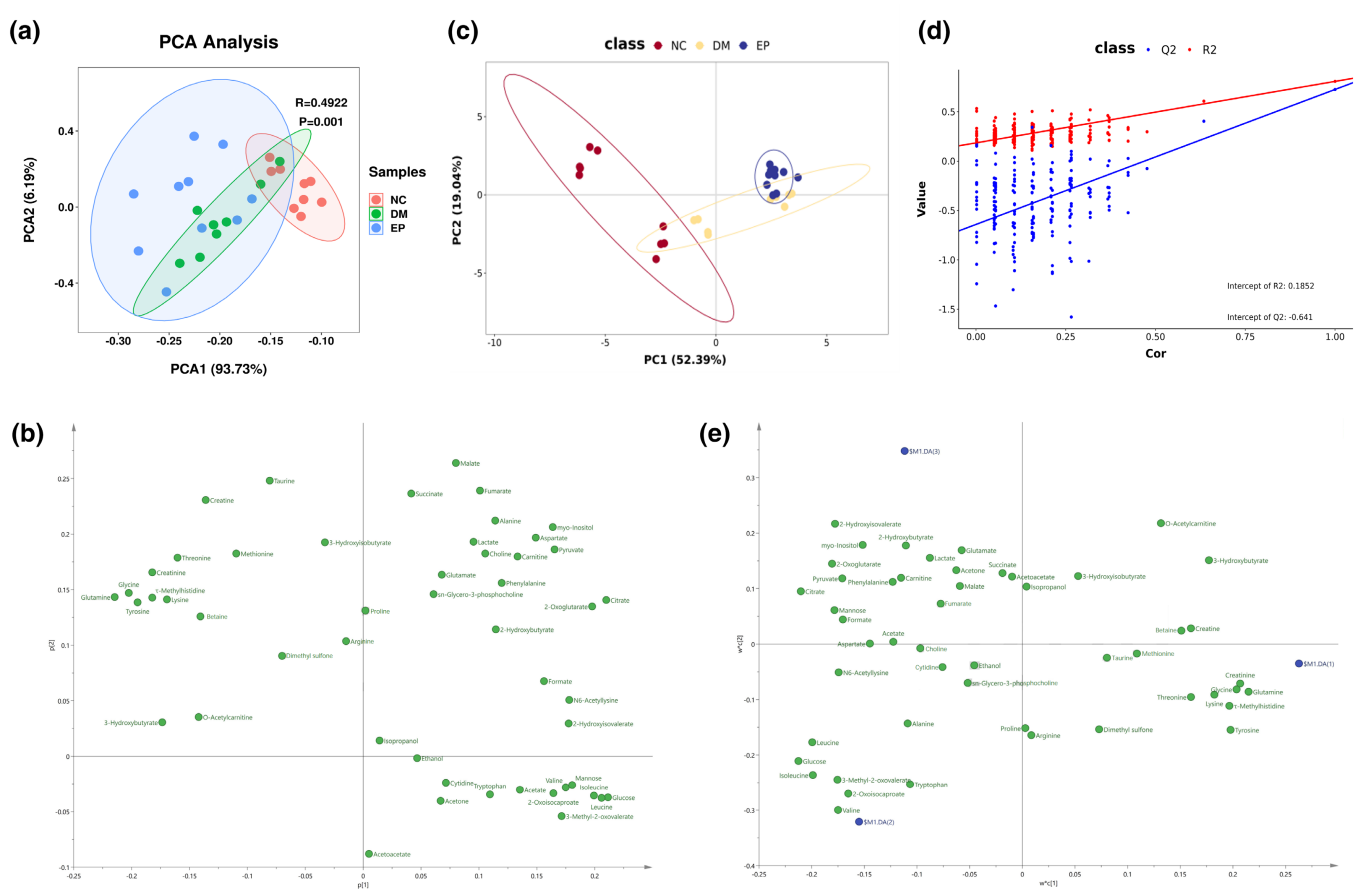
Principal component analysis (PCA) and PLS‐DA analysis of serum samples from three experimental groups. (a) PCA score plot. (b) Loading scatter plot of PCA. (c) PLS‐DA score plot. (d) Permutation test of PLS‐DA. (e) Loading scatter plot of PLS‐DA. NC: control group (*n* = 9); DM: type 2 diabetic model group (*n* = 8), EP: intervention group (*n* = 10), gavaged with 200 mg/kg body weight EP.

A representative ^1^H‐NMR spectrum of the metabolites detected in a serum sample is shown in Figure 2. From these spectra, 50 metabolites were identified, including 16 organic acids (2‐hydroxybutyrate, 2‐hydroxyisovalerate, 2‐oxoglutarate, 2‐oxoisocaproate, 3‐hydroxybutyrate, 3‐hydroxyisobutyrate, 3‐methyl‐2‐oxovalerate, acetoacetate, acetate, citrate, formate, fumarate, lactate, malate, pyruvate, and succinate), 22 amino acids and their derivatives (alanine, arginine, aspartate, betaine, carnitine, creatine, choline, glutamate, glutamine, glycine, isoleucine, leucine, lysine, methionine, O‐acetylcarnitine, phenylalanine, proline, taurine, threonine, tryptophan, tyrosine, valine), and 12 other metabolites. The chemical shifts and multiplicities of these identified metabolites are summarized in Table S3, and the quantification of serum metabolites in ZDF rats is shown in Table S4. Initially, an unsupervised PCA was performed on the mean‐centered and scaled NMR data of each experimental group. The results indicated a significant separation between the NC and DM groups, while the separation trend between the DM and EP groups was less pronounced (Figure 3a). The loading scatter plot of PCA (Figure 3b) showed that the separation of the groups was likely due to changes in succinate, fumarate, and malate. Subsequently, a supervised PLS‐DA with paretoscaled NMR data was applied to maximize the extent of information retrieval. The PLS‐DA score plots clearly demonstrated separation among the NC, DM, and EP groups (Figure 3c). The *R2Y* and *Q*2 values of the PLS‐DA models were 0.808 and 0.726, respectively. Figure 3d presents the validation results of 200 random permutation tests, which generated intercepts with *Q*2 = ‐0.641, indicating the model's reliability without overfitting (Chen et al., 2023; Li et al., 2019). The loading scatter plots of PLS‐DA revealed that valine, glucose, isoleucine, leucine, 3‐methyl‐2‐oxovalerate, 2‐oxoisocaproate, tryptophan, 3‐hydroxybutyrate, and O‐acetylcarnitine were the main contributors to the separation between groups (Figure 3e).

### 3.5 Identification of potential biomarkers in serum

**FIGURE 4:**
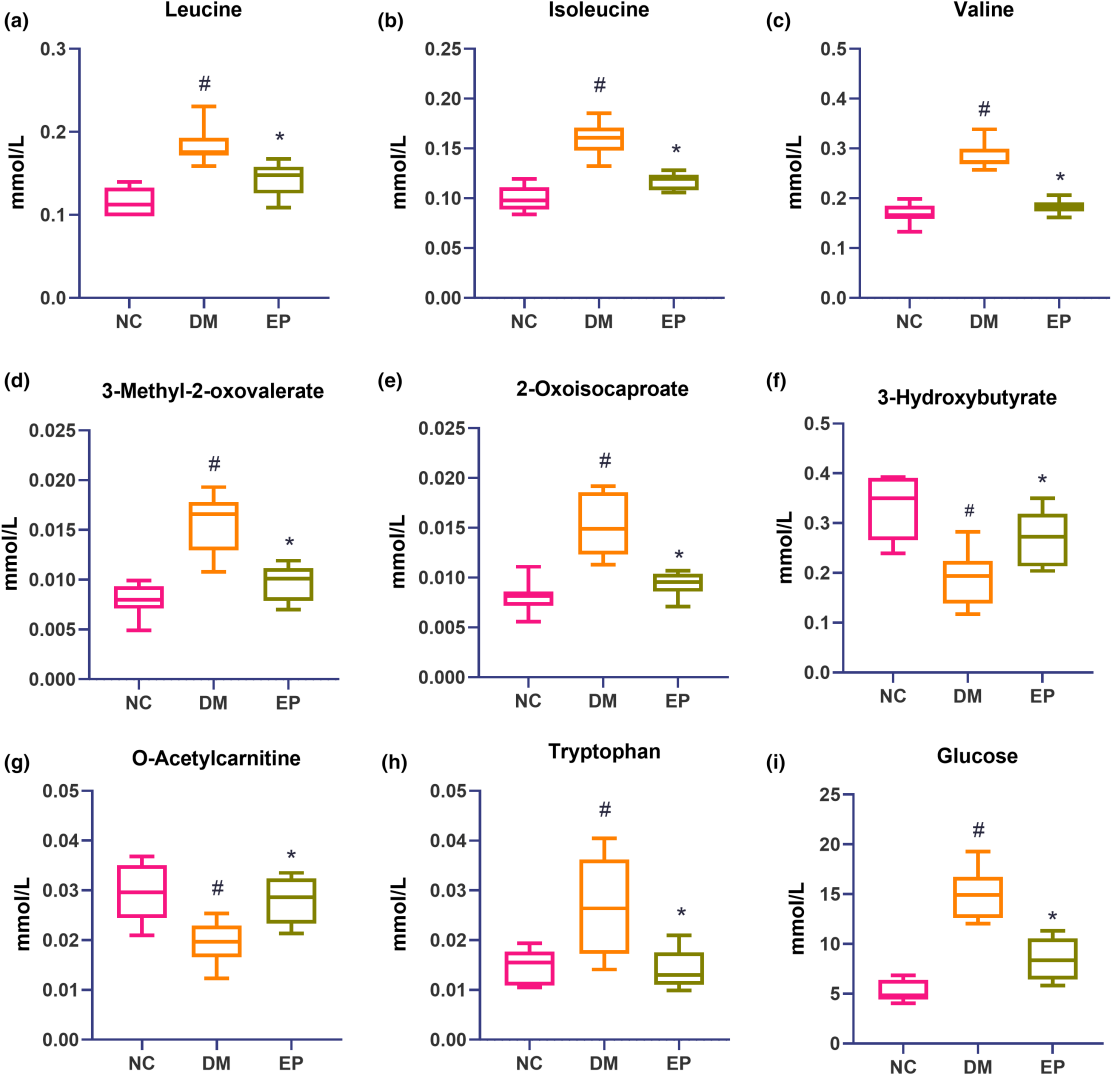
Box‐and‐whisker plots of differential metabolites among the three groups. (a) Leucine; (b) Isoleucine; (c) Valine; (d) 3‐Methyl‐2‐oxovalerate; (e) 2‐Oxoisocaproate; (f) 3‐Hydroxybutyrate; (g) O‐Acetylcarnitine; (h) Tryptophan; (i) Glucose. NC: control group (*n* = 9); DM: type 2 diabetic model group (*n* = 8), EP: intervention group (*n* = 10), gavaged with 200 mg/kg body weight EP. ^#^ *p* < .05 compared with the NC group; *p < .05 compared with the DM group.

Table 3 displays the fold change (FC) of differential metabolites according to the PLS‐DA model. Compared to those in the NC group, 17 metabolites were identified in the DM group, 14 of which were upregulated and 3 of which were downregulated. Treatment with EP significantly reversed the pattern of 9 out of the 17 metabolites. Consistently, Figure 4 demonstrates that leucine, isoleucine, valine, tryptophan, 3‐methyl‐2‐oxovalerate, 2‐oxoisocaproate, and glucose levels were markedly increased in the DM group, while 3‐hydroxybutyrate and O‐acetylcarnitine levels were markedly decreased. These metabolites showed significant reversal in the EP group, indicating the effective regulation of the metabolic network related to these metabolites by EP in diabetic mice.

**TABLE 3:** Quantitative comparison of serum metabolites in the NC, DM, and EP groups.

	Metabolites	DM/NC		EP/DM	
		*p‐*value	log_2_ (FC)	*p‐*value	log_2_ (FC)
1	3‐Hydroxybutyrate	.006	−0.74	.049	0.18
2	Acetylcarnitine	.002	−0.62	.005	0.3
3	Glycine	.016	−1.00	/	/
4	Alanine	.003	0.23	/	/
5	Aspartate	.002	0.37	/	/
6	Citrate	.001	0.42	/	/
7	Fumarate	.007	0.43	/	/
8	Acetate	.032	0.50	/	/
9	Leucine	.001	0.58	.009	−0.23
10	Isoleucine	.001	0.69	.001	−0.32
11	3‐Methyl‐2‐oxovalerate	.001	0.76	.044	−0.3
12	Mannose	.001	0.77	/	/
13	2‐Oxoisocaproate	.001	1.10	.022	−0.45
14	Glucose	.001	1.11	.049	−0.09
15	Tryptophan	.001	1.70	.007	−0.74
16	Valine	.001	1.75	.001	−0.3
17	2‐Hydroxyisovalerate	.013	3.22	.032	0.65

*Note*: Color coding according to log_2_ (FC), red represents an increase and blue represents a decrease. A darker color means the absolute value of log_2_ is greater than 1. The symbol “/” indicates that there are no significant differences in metabolites between the EP and DM groups. NC: control group (*n* = 9); DM: type 2 diabetic model group (*n* = 8), EP: intervention group (*n* = 10), gavaged with 200 mg/kg body weight EP.

### 3.6 Metabolic pathway and function analysis

**FIGURE 5:**
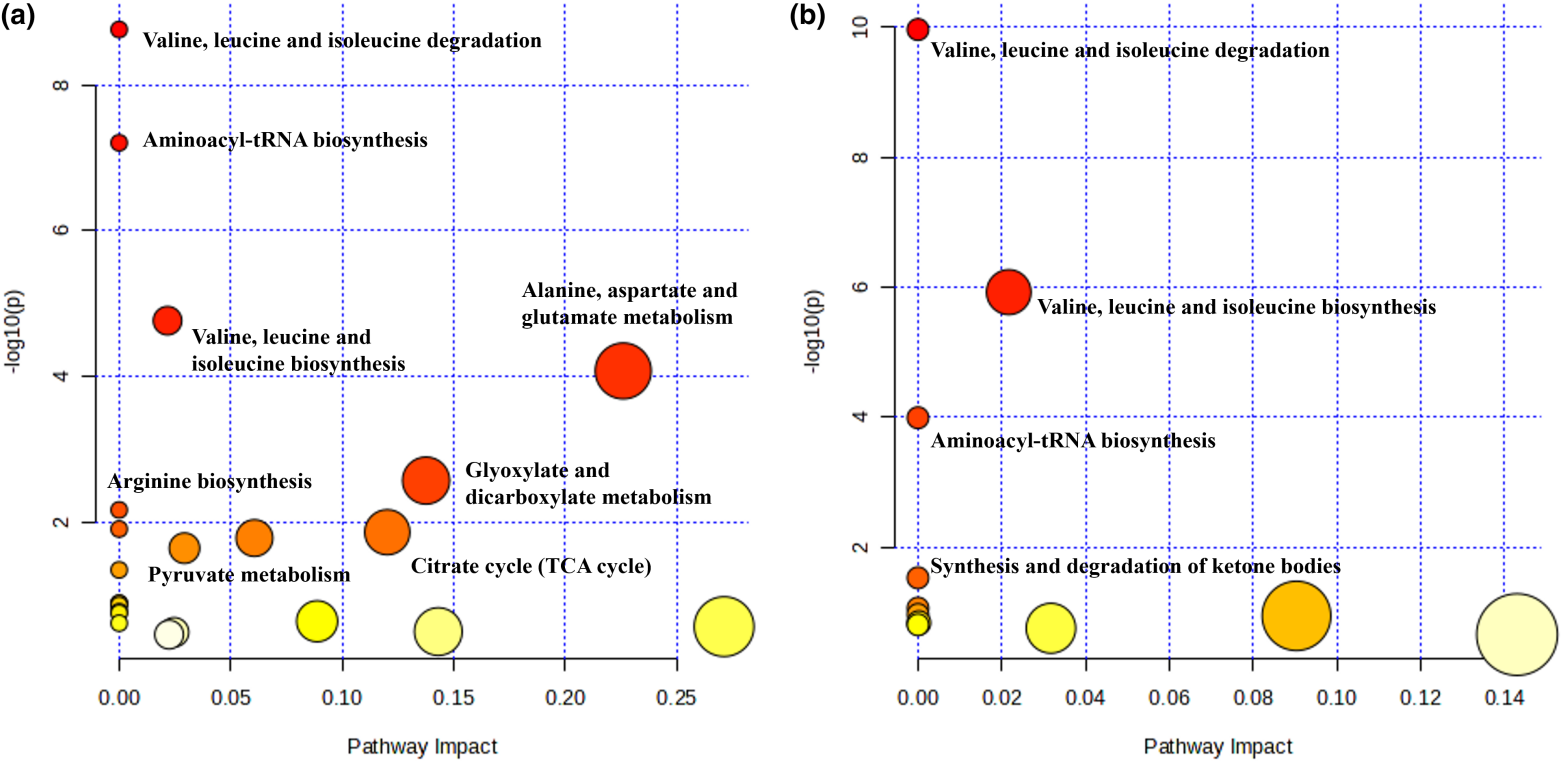
Metabolic pathway impact prediction based on the KEGG database. (a) Alterative pathway between the DM group and NC group. (b) Alterative pathway between the EP group and DM group. NC: control group (*n* = 9); DM: type 2 diabetic model group (*n* = 8), EP: intervention group (*n* = 10), gavaged with 200 mg/kg body weight EP.

**FIGURE 6:**
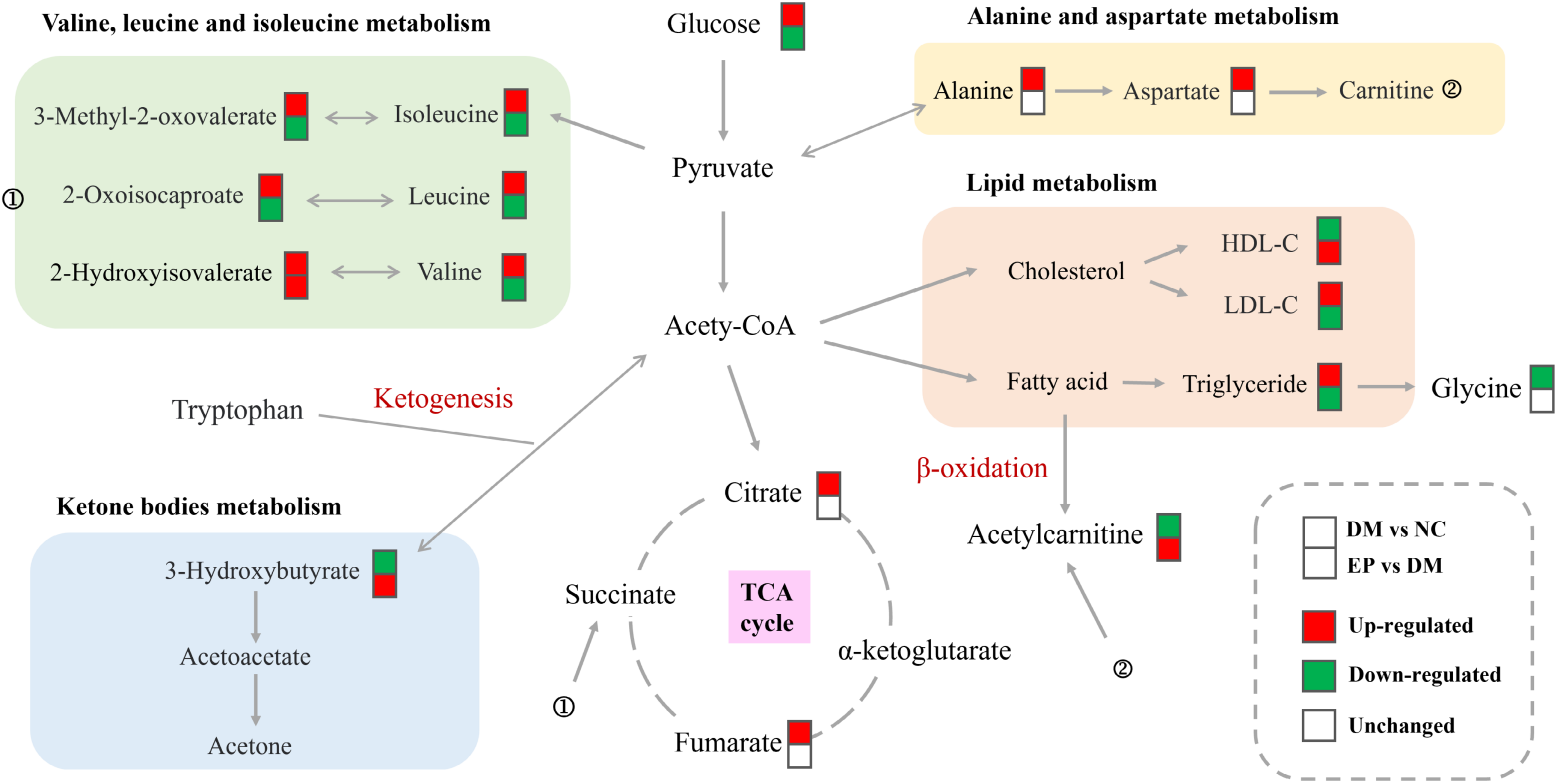
Summary of metabolic networks in diabetic and EP intervention rats. NC: control group (*n* = 9); DM: type 2 diabetic model group (*n* = 8), EP: intervention group (*n* = 10), gavaged with 200 mg/kg body weight EP.

The metabolic pathways were examined based on the different metabolites previously screened among the three groups, and the findings are illustrated in Figure 5. In comparison to those in the NC group, numerous pathways exhibited alterations in the DM group, with notable impacts on valine, leucine, and isoleucine synthesis; aminoacyl‐tRNA synthesis; valine, leucine, and isoleucine degradation; alanine, aspartate, and glutamate metabolism; and glyoxylate and dicarboxylate metabolism. Remarkably, EP intervention had the most significant influence on valine, leucine, and isoleucine synthesis; valine, leucine, and isoleucine degradation; aminoacyl‐tRNA synthesis; and the synthesis and degradation of ketone bodies. Based on relevant biomarkers and metabolic pathways, metabolite changes in response to diabetes and EP intervention, and the association networks between them are summarized in Figure 6.

### 3.7 Correlation analysis between metabolites and phenotypes

**FIGURE 7:**
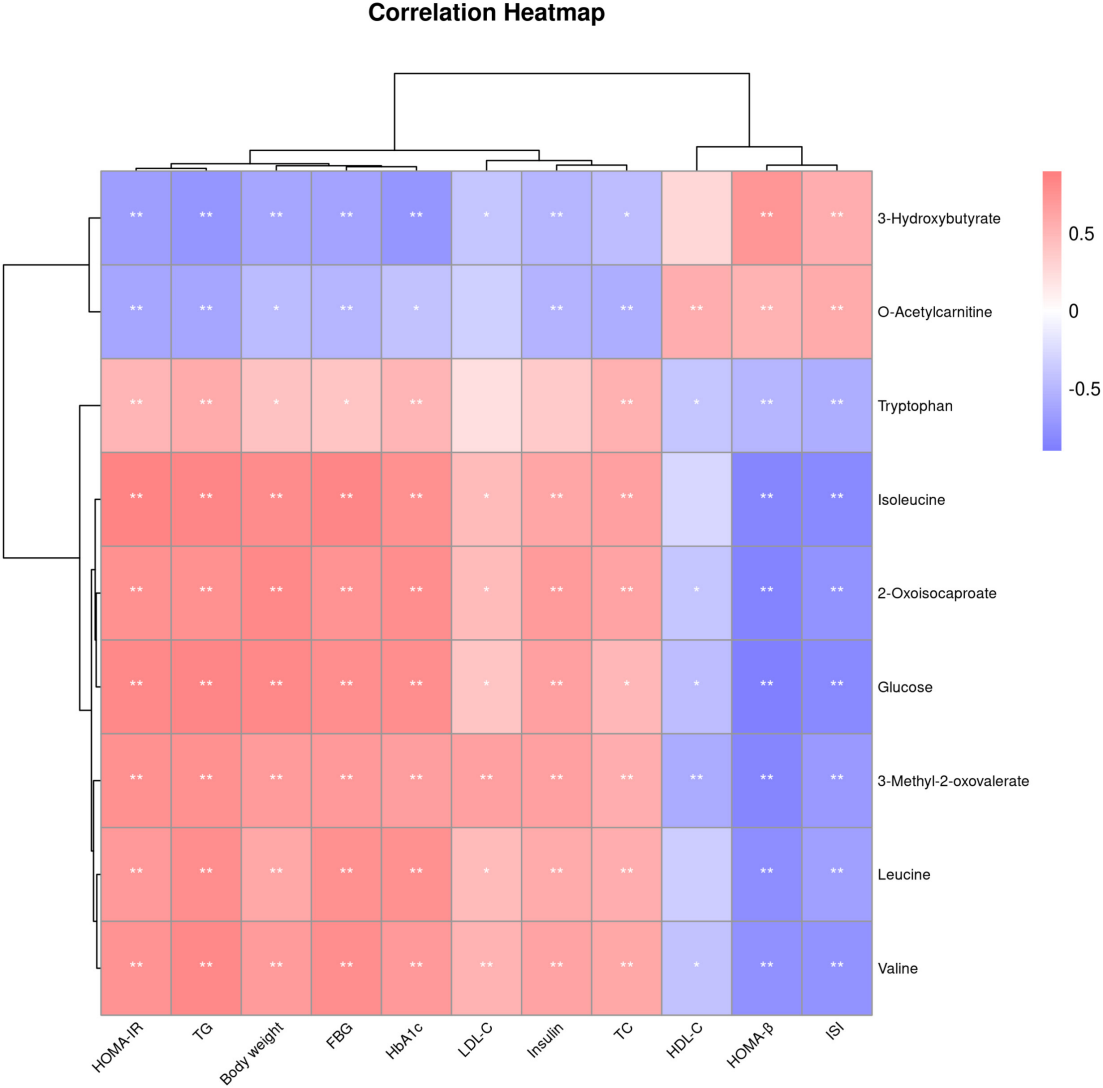
Correlation analysis between metabolites and phenotypes of rats. NC: control group (*n* = 9); DM: type 2 diabetic model group (*n* = 8), EP: intervention group (*n* = 10), gavaged with 200 mg/kg body weight EP. *p < .05, ** *p* < .01.

Correlations between candidate biomarkers and the phenotype of the rats were visualized through a heatmap based on the Spearman correlation analysis. Figure 7 shows that five metabolites from the valine, leucine, and isoleucine metabolic pathway were positively correlated with body weight, FBG, HbA1c, insulin levels, and HOMA‐IR, and negatively correlated with HOMA‐β and ISI. In contrast, 3‐hydroxybutyrate from the ketone body metabolic pathway and O‐acetylcarnitine from the fatty acid oxidation pathway were positively correlated with HOMA‐β and ISI, and negatively correlated with body weight, FBG, HbA1c, insulin levels, and HOMA‐IR.

## 4 DISCUSSION

*Enteromorpha prolifera* polysaccharide (EP) has been extensively studied for its various biological activities, such as anticoagulant, antioxidant, immunoregulatory, anti‐inflammatory, antitumor, antiaging, and hypolipidemic effects (Chen et al., 2022; Zhong et al., 2020). In this work, we investigated the hypoglycemic effect of EP in male Zucker diabetic fatty (ZDF) rats, which are known to be spontaneously obese and hyperglycemic due to defects in the leptin receptor and low inherited insulin promoter activity (Sultan et al., 2020). Our results showed that the EP group exhibited lower FBG, fasting insulin, and HbA1c levels than the DM group, indicating that EP effectively prevents the continuous increase in blood glucose levels associated with functional defects. EP has also been shown to enhance β‐cell function, increase insulin sensitivity, and improve insulin resistance caused by diabetes, as evidenced by a remarkable increase in HOMA‐β, ISI, and a decrease in HOMA‐IR. Additionally, the diabetic rats exhibited significant increases in serum lipid levels, which were reduced by EP treatment, suggesting improvement of dyslipidemia. It was noted that the effects of EP on different visceral fats are different. Currently, some studies have proposed that perirenal fat can be used as a potential diagnostic marker in patients with T2D (Guo et al., 2022). Our study found that EP has a significant effect on perirenal fat, which may bring more applications to biological efficacy of EP. Unlike the results for pancreatic mass, the effect of EP on liver mass was also not significant. This suggests that EP may be more inclined to act on the pancreas, preventing persistent blood glucose increases by improving pancreatic function.

Although several plant polysaccharides, such as *Dendrobium officinale* polysaccharide (Liu et al., 2020), *Pueraria lobata* root polysaccharide (Luo et al., 2021), and *Grifola frondosa* polysaccharide (Chen et al., 2018), have been reported to lower blood glucose levels, the metabolic processes of polysaccharides and the effects of their metabolites need to be evaluated more fully. Therefore, we used ^1^H NMR‐based metabolomics methods to investigate whether EP exerts an antidiabetic effect by altering the metabolic process in ZDF mice. This approach focuses on analyzing small and low‐mass molecules that are end products of biological metabolic pathways. Through our analysis, we identified nine biomarkers that could be indicative of the antidiabetic effects of EP, which were identified as being associated with branched‐chain amino acids (BCAAs) metabolism, ketone body metabolism, and fatty acid oxidation.

Branched‐chain amino acids (BCAAs) are composed of isoleucine, leucine, and valine, and are widely distributed in protein‐rich food sources consumed by humans. Numerous randomized controlled trials (RCTs) and prospective studies have consistently reported a positive correlation between BCAAs and the emergence of T2D (Ramzan et al., 2022; Wan et al., 2023). Notably, isoleucine, leucine, and valine are glycogenic amino acids that are degraded into glucose precursors, such as pyruvate, oxaloacetate, and α‐ketoglutaric acid. In the context of diabetes, the levels of glycogenic amino acids are reduced due to impaired glucose uptake in insulin‐resistant cells, reflecting the promotion of gluconeogenesis (Lu et al., 2020). In addition, some amino acids (such as valine) enter the tricarboxylic acid (TCA) cycle during insulin resistance to produce adenosine triphosphate (ATP) and energy for skeletal muscle (Li et al., 2020). Therefore, the degradation pathways of BCAAs are crucial for both energy and glucose metabolism. BCAAs are primarily catabolized in mitochondria through the involvement of BCAA aminotransferase and branched‐chain α‐ketoacid dehydrogenase. Increased BCAAs degradation results in increased BCAAs catabolism intermediates, which may disrupt mitochondrial glucose oxidation, leading to mitochondrial stress and impaired insulin secretion (Zheng et al., 2022). 3‐Methyl‐2‐oxovalerate, a branched ketoacid derivative of isoleucine, was identified as the strongest predictor of impaired fasting glucose in a clinical study (Menni et al., 2013). Similarly, 2‐oxoisocaproate, an intermediate of leucine metabolism, was found to be closely associated with pathoglycemia caused by insulin resistance and diabetes (Stec et al., 2015). In our findings, serum concentrations of 3‐methyl‐2‐oxovalerate, 2‐oxoisocaproate, leucine, isoleucine, and valine in the EP group were markedly lower than those in the DM group, indicating that the disorder of BCAAs metabolism caused by diabetes might be partially recovered by EP treatment.

3‐Hydroxybutyrate is one of the main ketone bodies in the body and is produced by the liver from fatty acids during fasting or prolonged strenuous exercise. It is synthesized from acetyl coenzyme A (acetyl‐CoA) and serves as an essential carrier of energy from the liver to extrahepatic tissues, particularly when the glucose supply is insufficient (Fukao et al., 2014). T2D has been associated with disturbances in ketone body metabolism, characterized by reduced ketone body oxidation and CoA transferase function (Cotter et al., 2013). Animal studies have demonstrated a negative correlation between fasting blood glucose and hydroxybutyric acid levels, which may be caused by decreased ketogenic ability (Hu et al., 2022). In the present study, the serum 3‐hydroxybutyrate level in diabetic rats was markedly lower than that in normal rats, indicating a disorder of ketone body metabolism. Notably, a clinical study showed that higher serum 3‐hydroxybutyrate levels were associated with a better response to antidiabetic therapy and improvement in hyperglycemia (Lee et al., 2023). Our results showed that serum 3‐hydroxybutyrate levels were significantly increased after EP intervention, supporting its beneficial role in antidiabetes. Consistent results have been reported in a study investigating the antidiabetic mechanism of *Berberis vernae* (Li et al., 2020). Tryptophan is an essential amino acid crucial for protein biosynthesis. It must be acquired through the diet since mammalian cells lack the gene encoding tryptophan synthase (Sudar‐Milovanovic et al., 2022). Numerous clinical studies have consistently reported markedly higher serum tryptophan levels in diabetic patients than in nondiabetic patients, especially regarding the 1‐year changes in tryptophan concentrations in people with diabetes, which may be attributed to increased initial compensation (Chen et al., 2016; Yu et al., 2018). Our study showed consistent results, as the serum tryptophan level in the DM group was significantly higher than that in the NC group. This abnormal increase was reversed by EP, demonstrating that the prevention of diabetes by EP was partially dependent on changes in serum tryptophan concentrations. Moreover, tryptophan is a ketogenic amino acid that is metabolized to be degraded into acetyl‐CoA or acetoacetic acid and can therefore be converted into fatty acids or ketone bodies (Zheng et al., 2022). In this work, the decrease in the serum tryptophan levels observed in the EP group was consistent with its degradation and accompanying increase in 3‐hydroxybutyrate levels. Collectively, EP exerts antidiabetic effects by partially regulating ketone body metabolism pathways, including the synthesis and degradation of ketone bodies, and the conversion of ketogenic amino acids.

Acylcarnitine is a fatty acid metabolite involved in the metabolism of triglycerides, cholesterol, and other lipids. Free carnitine binds to the acetyl‐CoA produced by the β‐oxidation of fatty acids and is converted to acetylcarnitine by carnitine acetyltransferase (Zhao et al., 2021). Furthermore, acylcarnitine also participates in the process of gluconeogenesis, which reflects changes in glucose metabolism in diabetic patients (Zhao et al., 2021). Previous studies have demonstrated that acetylcarnitine is the most abundant single acyl‐L‐carnitine, and this metabolite is significantly increased in T2D patients, reaching approximately 1.57‐fold of the level in nondiabetic patients (Adams et al., 2009). However, in our study, the serum acetylcarnitine concentration of rats in the DM group was markedly lower than that in the NC group, which was not consistent with the studies mentioned above. Notably, the concentration of acetylcarnitine in the blood represents the total amount in the whole body, primarily derived from skeletal muscle and the brain (Klepochova et al., 2020). Skeletal muscle accounts for utilizing 80% of the body's glucose intake and plays a pivotal role in regulating glucose balance (Ciaraldi et al., 2010), while also serving as the primary site for acetylcarnitine synthesis. In terms of skeletal muscle glucose metabolism, a restricted ability to produce acetylcarnitine might reduce the activity of pyruvate dehydrogenase, resulting in a decrease in glycolysis, which is a major cause of skeletal muscle insulin resistance (Klepochova et al., 2020). It has been confirmed that the reduced acetylcarnitine formation in the musculi soleus of T2D patients may be the cause of reduced insulin sensitivity, reflecting chronic hyperglycemia (Lindeboom et al., 2014). These findings provide an explanation for the observed decrease in the serum acetylcarnitine concentration in our diabetic rats. Interestingly, acetylcarnitine has been identified as a beneficial metabolite in lowering blood lipids. A study on probiotics revealed that acetylcarnitine levels increased significantly following probiotic supplementation, resulting in decreased serum TC and TG levels (Huan Wang et al., 2023). Consistent effects were also observed with EP, including an increase in the serum acetylcarnitine concentration and a decrease in levels of TC and TG. These findings suggest that acetylcarnitine contributes to the antidiabetic effect of EP and has beneficial effects on blood lipid levels.

Due to the β (1‐4) glycosidic bond, most polysaccharides derived from green alga may not be digestible by humans. These polysaccharides pass through the small intestine unmetabolized and are fermented by intestinal bacteria into short‐chain fatty acids (SCFAs) (Hemsworth et al., 2016). According to the results of in vitro fermentation experiments, the levels of acetic, butyric, and lactic acids increase significantly after EP is fermented by the human fecal microbiota (Kong et al., 2016). Studies have shown that *Ulva* polysaccharide is not destroyed in the human digestive system but is selectively absorbed by some organs, with no obvious signs of harm to normal cells (Thanh et al., 2016). These findings suggest that EP can be utilized by the intestinal microbiome as a potential mediator involved in the regulation of gut function. The biological activity of polysaccharides is mostly related to their dose. A clinical study was conducted in which 2 g/day *Ulva* polysaccharide was administered orally to 64 overweight or obese participants (Roach et al., 2022). Additionally, according to the National Nutrition Survey of Japan, the daily intake of algal polysaccharides by adults is approximately 14.3 g. In the present study, the intragastric dose for rats was 200 mg/kg. According to the dose factor method (Nair & Jacob, 2016), the human equivalent dose (HED) of EP was calculated using the correction factor as follows: HED = 200 mg/kg × (6/37) = 32.4 mg/kg. In light of this, an adult weighing 70 kg would receive a dose of 2.3 g/d EP. This dose is consistent with the clinical trials described above. Although there are no data on the absorption, distribution, or excretion of EP in the human body, several active carbohydrate enzymes have been reported to hydrolyze *Ulva* polysaccharides (Pradhan et al., 2023). More clinical studies and pharmacokinetic studies will be performed in the future to determine the efficacy of EP as an antidiabetic drug.

## 5 CONCLUSIONS

After 9 weeks of intervention, EP effectively prevented blood glucose abnormalities caused by leptin receptor defects in ZDF rats. Moreover, EP significantly reduced serum lipid levels, suggesting improvement of dyslipidemia. ^1^H NMR‐based metabolomics analysis revealed that the concentrations of metabolites in the serum of diabetic mice were altered by EP, and nine different metabolites were screened as biomarkers. Metabolic pathway enrichment analysis revealed that the antidiabetic mechanism of EP relies on regulating the disorder of BCAA metabolism, aminoacyl‐tRNA biosynthesis, and ketone body metabolism. These findings will provide a theoretical basis for further research on the mechanism of the hypoglycemic and antidiabetic effects of green algae polysaccharides.

## AUTHOR CONTRIBUTIONS

**Jie Chen:** Data curation (equal); software (equal); writing – original draft (equal). **Shuting Wang:** Methodology (equal); software (equal); visualization (equal). **Fuchuan Guo:** Project administration (equal); resources (equal); supervision (equal). **Yupeng Gong:** Data curation (equal); software (equal); visualization (equal). **Tianbao Chen:** Formal analysis (equal); project administration (equal). **Chris Shaw:** Resources (equal); supervision (equal). **Rencai Jiang:** Software (equal); visualization (equal). **Fang Huang:** Conceptualization (equal); formal analysis (equal); supervision (equal). **Dai Lin:** Conceptualization (equal); investigation (equal); writing – review and editing (equal).

## FUNDING INFORMATION

This work was supported by the Natural Science Foundation of Fujian Province (No. 2021J01724), the Startup Fund for scientific research of Fujian Medical University (No. 2019QH1001), and the Young and Middle‐aged Teacher Education Research Project of Fujian Province (JAT200113).

## CONFLICT OF INTEREST STATEMENT

The authors declare that they have no known competing financial interests or personal relationships that could have appeared to exert influence on the research presented in this manuscript.

## ETHICS STATEMENT

The experimental protocols adhered to the guidelines outlined in the National Research Council's Guide for the Care and Use of Laboratory Animals. Approval for the experimental procedures was obtained from the Animal Care and Use Committee of Fujian Medical University (Approval No. FJMU 2021‐0012).

## Supporting information


Table S1.

## Data Availability

The data supporting the findings of this study are available from the authors upon reasonable request.
