# Supplementary material for: 1H‐NMR‐based metabolomics reveals the preventive effect of Enteromorpha prolifera polysaccharides on diabetes in Zucker diabetic fatty rats
Source: Food Sci Nutr. 2024 Mar 5;12(6):4049–62. doi: 10.1002/fsn3.4061 (PMC11167149; doi:10.1002/fsn3.4061)
Supplement: Supplementary file 1 — Table S1. [file FSN3-12-4049-s001.docx]

TABLES

Table S1 The sensitivity and detection range of commercial kits.

| Commercial kits | Detection range | Sensitivity |
| --- | --- | --- |
| TG kits | 0.05~9.0 mmol/L | 2.26 mmol/L concentration calibration product, △A ≥0.0700 Abs |
| TC kits | 0~19.39 mmol/L | 0.5 mmol/L concentration calibration product, △A is 0.01~0.08 Abs |
| HDL-C kits | 0~5.16 mmol/L | 1.0 mmol/L concentration calibration product, △A ≥0.04 Abs |
| LDL-C kits | 0~10.4 mmol/L | 2.6mmol/L concentration calibration product, △A is 0.180 ~ 0.280 Abs |
| Insulin ELISA kits | 3~200 mU/L | ≤ 3 mU/L |
| HbA1c ELISA kits | 1.563~100 ng/mL | 0.938 ng/mL |

Table S2 Chemical composition of Purina #5008 diet.

| Nutrients | Purina #5008 diet |
| --- | --- |
| **Protein, %** | **23.5** |
| Arginine, % | 1.44 |
| Cystine, % | 0.35 |
| Glycine, % | 1.23 |
| Histidine, % | 0.58 |
| Isoleucine, % | 1.20 |
| Leucine, % | 1.87 |
| Lysine, % | 1.40 |
| Methionine, % | 0.43 |
| Phenylalanine, % | 1.08 |
| Tyrosine, % | 0.66 |
| Threonine, % | 0.90 |
| Tryptophan, % | 0.28 |
| Valine, % | 1.19 |
| Serine, % | 1.20 |
| Aspartic Acid, % | 2.60 |
| Glutamic Acid, % | 4.77 |
| Alanine, % | 1.39 |
| Proline, % | 1.63 |
| Taurine, % | 0.02 |
| **Fat (acid hydrolysis), %** | **7.5** |
| Cholesterol, ppm | 280 |
| Linoleic Acid, % | 1.37 |
| Linolenic Acid, % | 0.09 |
| Arachidonic Acid, % | 0.01 |
| Table S2 (Continued) |  |
| Omega-3 Fatty Acid, % | 0.29 |
| **Fiber (Crude), %** | **3.8** |
| **Ash, %** | **6.8** |
| Calcium, % | 1.00 |
| Phosphorus, % | 0.65 |
| Phosphorus(non-phytate), % | 0.42 |
| Potassium, % | 1.10 |
| Magnesium, % | 0.20 |
| Sulfur, % | 0.24 |
| Sodium, % | 0.28 |
| Chloride, % | 0.48 |
| Fluorine, ppm | 19 |
| Iron, ppm | 230 |
| Zinc, ppm | 73 |
| Manganese, ppm | 71 |
| Copper, ppm | 13 |
| Cobalt, ppm | 0.4 |
| Iodine, ppm | 0.8 |
| Chromium, ppm | 1.4 |
| Selenium, ppm | 0.23 |
| Carotene, ppm | 4.0 |
| Vitamin K (as menadione), ppm | 3.2 |
| Thiamin Hydrochloride, ppm | 16 |
| Riboflavin, ppm | 5.0 |
| Niacin, ppm | 109 |
| Pantothenic Acid, ppm | 15 |
| Choline Chloride, ppm | 2000 |
| Folic Acid, ppm | 3.0 |
| Pyridoxine, ppm | 6.0 |
| Biotin, ppm | 0.20 |
| B_12_, mcg/kg | 20 |
| Vitamin A, IU/gm | 15 |
| Vitamin D_3_ (added), IU/gm | 3.3 |
| Vitamin E, IU/kg | 55 |

Table S3 Chemical shifts and multiplicities for metabolites identified in a serum by ^1^H NMR (corresponding to Figure 2).

| No. | Metabolites | Chemical shift (ppm) | No. | Metabolites | Chemical shift (ppm) |
| --- | --- | --- | --- | --- | --- |
| 1 | 2-Hydroxybutyrate | 0.88(t) | 26 | Glutamate | 2.00(m), 2.41(m) |
| 2 | 2-Hydroxyisovalerate | 0.82(d) | 27 | Glutamine | 2.13(m) |
| 3 | 2-Oxoglutarate | 3.00(t) | 28 | Glycine | 3.56(s) |
| Table S3 (Continued) | | | | | |
| 4 | 2-Oxoisocaproate | 0.93(d) | 29 | Isoleucine | 0.93(t), 1.00(d) |
| 5 | 3-Hydroxybutyrate | 1.19(d) | 30 | Isopropanol | 1.18(t) |
| 6 | 3-Hydroxyisobutyrate | 1.11(d) | 31 | Lactate | 1.32(d), 4.09(q) |
| 7 | 3-Methyl-2-oxovalerate | 0.88(t) | 32 | Leucine | 0.96(d) |
| 8 | Acetate | 1.92(s) | 33 | Lysine | 1.70(m), 1.88(m), 3.01(t) |
| 9 | Acetoacetate | 2.27(s) | 34 | Malate | 2.67(dd) |
| 10 | Acetone | 2.22(s) | 35 | Mannose | 4.88(d) |
| 11 | Alanine | 1.47(d) | 36 | Methionine | 2.65(dd) |
| 12 | Arginine | 1.69(m), 1.90(m) | 37 | N6-Acetyllysine | 2.02(s) |
| 13 | Aspartate | 2.80(dd) | 38 | O-Acetylcarnitine | 2.18(s) |
| 14 | Betaine | 3.25(s), 3.89(s) | 39 | Phenylalanine | 7.42(t) |
| 15 | Carnitine | 3.22(s) | 40 | Proline | 1.99(m) |
| 16 | Choline | 3.19(s) | 41 | Pyruvate | 2.35(s) |
| 17 | Citrate | 2.52(d), 2.66(d) | 42 | Succinate | 2.43(s) |
| 18 | Creatine | 3.02(s), 3.92(s) | 43 | Taurine | 3.27(t) |
| 19 | Creatinine | 3.04(s) | 44 | Threonine | 4.24(m) |
| 20 | Cytidine | 6.04(d), 7.83(d) | 45 | Tryptophan | 7.55(d) |
| 21 | Dimethyl sulfone | 3.14(s) | 46 | Tyrosine | 6.88(m),7.17(m) |
| 22 | Ethanol | 1.18(t) | 47 | Valine | 0.98(d), 1.03(d) |
| 23 | Formate | 8.44(s) | 48 | myo-Inositol | 3.33(t), 3.62(t) |
| 24 | Fumarate | 6.52(s) | 49 | sn-Glycero-3-phosphocholine | 3.22(s) |
| 25 | Glucose | 3.26(dd), 3.54(dd), 4.45(d), 5.24(d) | 50 | τ-Methylhistidine | 7.01(s), 7.77(s) |

s=singlet; d=doublet; dd=double doublet; t=triplet; q=quartet; m=multiplet.

Table S4 Identified and quantified serum metabolites in ZDF rats from ^1^H-NMR spectra.

| No. | Metabolic | Concentration(mmol/L) | | | | | | | | |
| --- | --- | --- | --- | --- | --- | --- | --- | --- | --- | --- |
|  |  | NC | | | MD | | | EP | | |
| 1 | 2-Hydroxybutyrate | 0.00555 | ± | 0.00000 | 0.00683 | ± | 0.00000 | 0.00902 | ± | 0.00001 |
| 2 | 2-Hydroxyisovalerate | 0.00030 | ± | 0.00000 | 0.00280 | ± | 0.00001 | 0.00534 | ± | 0.00000 |
| 3 | 2-Oxoglutarate | 0.05219 | ± | 0.00021 | 0.07673 | ± | 0.00028 | 0.08884 | ± | 0.00025 |
| 4 | 2-Oxoisocaproate | 0.00809 | ± | 0.00000 | 0.01518 | ± | 0.00001 | 0.00936 | ± | 0.00000 |
| 5 | 3-Hydroxybutyrate | 0.33327 | ± | 0.00407 | 0.18986 | ± | 0.00274 | 0.27306 | ± | 0.00297 |
| 6 | 3-Hydroxyisobutyrate | 0.02902 | ± | 0.00004 | 0.02541 | ± | 0.00001 | 0.02900 | ± | 0.00003 |
| 7 | 3-Methyl-2-oxovalerate | 0.00798 | ± | 0.00000 | 0.01541 | ± | 0.00001 | 0.00963 | ± | 0.00000 |
| 8 | Acetate | 0.05361 | ± | 0.00018 | 0.07574 | ± | 0.00037 | 0.07300 | ± | 0.00077 |
| 9 | Acetoacetate | 0.06027 | ± | 0.00127 | 0.05284 | ± | 0.00050 | 0.07599 | ± | 0.00218 |
| 10 | Acetone | 0.01993 | ± | 0.00007 | 0.02240 | ± | 0.00003 | 0.03045 | ± | 0.00035 |
| Table S4 (Continued) | | | | | | | | | | |
| 11 | Alanine | 0.60364 | ± | 0.00554 | 0.70740 | ± | 0.00455 | 0.63147 | ± | 0.00720 |
| 12 | Arginine | 0.19780 | ± | 0.00217 | 0.21067 | ± | 0.00105 | 0.17562 | ± | 0.00141 |
| 13 | Aspartate | 0.06251 | ± | 0.00019 | 0.08060 | ± | 0.00021 | 0.07804 | ± | 0.00016 |
| 14 | Betaine | 0.16338 | ± | 0.00080 | 0.12960 | ± | 0.00038 | 0.13776 | ± | 0.00038 |
| 15 | Carnitine | 0.04918 | ± | 0.00012 | 0.05528 | ± | 0.00003 | 0.05993 | ± | 0.00007 |
| 16 | Choline | 0.02795 | ± | 0.00003 | 0.03146 | ± | 0.00001 | 0.03077 | ± | 0.00001 |
| 17 | Citrate | 0.22860 | ± | 0.00247 | 0.30634 | ± | 0.00016 | 0.32002 | ± | 0.00070 |
| 18 | Creatine | 0.31348 | ± | 0.00599 | 0.22744 | ± | 0.00097 | 0.24897 | ± | 0.00135 |
| 19 | Creatinine | 0.03103 | ± | 0.00004 | 0.02089 | ± | 0.00000 | 0.01999 | ± | 0.00001 |
| 20 | Cytidine | 0.02943 | ± | 0.00001 | 0.03207 | ± | 0.00002 | 0.03092 | ± | 0.00001 |
| 21 | Dimethyl sulfone | 0.01162 | ± | 0.00001 | 0.01088 | ± | 0.00001 | 0.00850 | ± | 0.00001 |
| 22 | Ethanol | 0.01949 | ± | 0.00003 | 0.02314 | ± | 0.00008 | 0.02108 | ± | 0.00009 |
| 23 | Formate | 0.02123 | ± | 0.00002 | 0.02862 | ± | 0.00005 | 0.02882 | ± | 0.00001 |
| 24 | Fumarate | 0.00576 | ± | 0.00001 | 0.00692 | ± | 0.00000 | 0.00767 | ± | 0.00001 |
| 25 | Glucose | 5.21983 | ± | 1.12699 | 14.39896 | ± | 3.59729 | 8.59326 | ± | 4.23537 |
| 26 | Glutamate | 0.19112 | ± | 0.00170 | 0.19427 | ± | 0.00053 | 0.23270 | ± | 0.00335 |
| 27 | Glutamine | 0.71412 | ± | 0.02552 | 0.42623 | ± | 0.00896 | 0.38812 | ± | 0.00632 |
| 28 | Glycine | 0.33815 | ± | 0.01515 | 0.16973 | ± | 0.00089 | 0.14724 | ± | 0.00206 |
| 29 | Isoleucine | 0.09946 | ± | 0.00014 | 0.15964 | ± | 0.00025 | 0.11731 | ± | 0.00007 |
| 30 | Isopropanol | 0.00354 | ± | 0.00000 | 0.00334 | ± | 0.00000 | 0.00374 | ± | 0.00000 |
| 31 | Lactate | 8.83943 | ± | 11.92744 | 9.95118 | ± | 2.95479 | 12.28496 | ± | 11.23900 |
| 32 | Leucine | 0.11604 | ± | 0.00027 | 0.18401 | ± | 0.00054 | 0.14178 | ± | 0.00040 |
| 33 | Lysine | 0.43225 | ± | 0.00490 | 0.35081 | ± | 0.00126 | 0.33341 | ± | 0.00087 |
| 34 | Malate | 0.04146 | ± | 0.00041 | 0.04563 | ± | 0.00004 | 0.05408 | ± | 0.00043 |
| 35 | Mannose | 0.06828 | ± | 0.00016 | 0.11647 | ± | 0.00121 | 0.12061 | ± | 0.00120 |
| 36 | Methionine | 0.06197 | ± | 0.00005 | 0.05664 | ± | 0.00003 | 0.05690 | ± | 0.00003 |
| 37 | N6-Acetyllysine | 0.01091 | ± | 0.00001 | 0.01608 | ± | 0.00001 | 0.01447 | ± | 0.00000 |
| 38 | O-Acetylcarnitine | 0.02962 | ± | 0.00003 | 0.01954 | ± | 0.00002 | 0.02820 | ± | 0.00002 |
| 39 | Phenylalanine | 0.06492 | ± | 0.00007 | 0.07239 | ± | 0.00003 | 0.07696 | ± | 0.00015 |
| 40 | Proline | 0.16929 | ± | 0.00077 | 0.17732 | ± | 0.00075 | 0.15795 | ± | 0.00019 |
| 41 | Pyruvate | 0.27682 | ± | 0.00575 | 0.36459 | ± | 0.00123 | 0.39842 | ± | 0.00416 |
| 42 | Succinate | 0.04117 | ± | 0.00087 | 0.03583 | ± | 0.00009 | 0.05951 | ± | 0.00278 |
| 43 | Taurine | 0.36734 | ± | 0.03010 | 0.29059 | ± | 0.00688 | 0.28656 | ± | 0.01298 |
| 44 | Threonine | 0.28342 | ± | 0.00905 | 0.19469 | ± | 0.00258 | 0.16873 | ± | 0.00199 |
| 45 | Tryptophan | 0.01462 | ± | 0.00001 | 0.02659 | ± | 0.00009 | 0.01411 | ± | 0.00001 |
| 46 | Tyrosine | 0.12261 | ± | 0.00033 | 0.09679 | ± | 0.00013 | 0.08446 | ± | 0.00008 |
| 47 | Valine | 0.16921 | ± | 0.00039 | 0.28358 | ± | 0.00064 | 0.18361 | ± | 0.00018 |
| 48 | myo-Inositol | 0.06722 | ± | 0.00026 | 0.08057 | ± | 0.00016 | 0.09328 | ± | 0.00007 |
| 49 | sn-Glycero-3-phosphocholine | 0.02515 | ± | 0.00009 | 0.02953 | ± | 0.00004 | 0.02626 | ± | 0.00004 |
| 50 | τ-Methylhistidine | 0.05572 | ± | 0.00010 | 0.04229 | ± | 0.00002 | 0.03869 | ± | 0.00003 |

Data are presented as means ± SD. NC: control group (n=9); DM: type 2 diabetic model group (n=8), EP: intervention group (n=10), gavaged with 200 mg/kg body weight EP.
